# Multiple conserved regulatory domains promote *Fezf2* expression in the developing cerebral cortex

**DOI:** 10.1186/1749-8104-9-6

**Published:** 2014-03-12

**Authors:** Matthew J Eckler, Kathryn A Larkin, William L McKenna, Sol Katzman, Chao Guo, Robin Roque, Axel Visel, John L R Rubenstein, Bin Chen

**Affiliations:** 1 Department of Molecular, Cell and Developmental Biology, University of California, Santa Cruz, CA, USA; 2 Center for Biomolecular Science and Engineering, University of California, Santa Cruz, CA, USA; 3 Genomics Division, MS 84–171, Lawrence Berkeley National Laboratory, Berkeley, CA, USA; 4 U.S. Department of Energy Joint Genome Institute, Walnut Creek, CA, USA; 5 Nina Ireland Laboratory of Developmental Neurobiology, Department of Psychiatry, University of California, San Francisco, CA, USA

**Keywords:** *Fezf2*, Enhancer, Promoter, Cerebral cortex, Gene regulation, Transcription

## Abstract

**Background:**

The genetic programs required for development of the cerebral cortex are under intense investigation. However, non-coding DNA elements that control the expression of developmentally important genes remain poorly defined. Here we investigate the regulation of *Fezf2*, a transcription factor that is necessary for the generation of deep-layer cortical projection neurons.

**Results:**

Using a combination of chromatin immunoprecipitation followed by high throughput sequencing (ChIP-seq) we mapped the binding of four deep-layer-enriched transcription factors previously shown to be important for cortical development. Building upon this we characterized the activity of three regulatory regions around the *Fezf2* locus at multiple stages throughout corticogenesis. We identified a promoter that was sufficient for expression in the cerebral cortex, and enhancers that drove reporter gene expression in distinct forebrain domains, including progenitor cells and cortical projection neurons.

**Conclusions:**

These results provide insight into the regulatory logic controlling *Fezf2* expression and further the understanding of how multiple non-coding regulatory domains can collaborate to control gene expression *in vivo*.

## Background

Current estimates suggest that greater than half of evolutionarily conserved sequences in the human genome do not correspond to protein coding regions [1-4]. Transcriptional enhancers, non-coding DNA sequences that promote gene expression in a spatial and temporal manner, constitute a substantial portion of these regions [5]. Genome-wide association studies (GWAS) have identified sequence variations within putative enhancers that are associated with a wide range of neurological disorders such as autism, epilepsy, and schizophrenia [6-10]. However, the function of these DNA elements during normal brain development remains poorly understood [11].

The cerebral cortex contains six layers of projection neurons that are generated in a stereotyped manner such that deep-layer neurons, layers 5 and 6 (L5 and L6), are born before upper-layer neurons [12]. It has previously been shown that the transcription factor *Fezf2* is necessary and sufficient for the generation of deep-layer subcerebral projection neurons (SCPNs) [13-16]. Collectively, these studies suggest that *Fezf2* functions high in a transcriptional hierarchy that regulates SCPN development and fate specification [14,17-19]. Despite the essential role of *Fezf2* in neural development, little is known about how its transcription is precisely regulated.

To investigate the regulatory mechanisms controlling *Fezf2* transcription, we utilized chromatin immunoprecipitation combined with high throughput DNA sequencing (ChIP-seq) to identify a transcription factor-binding signature around the *Fezf2* locus. Guided by our ChIP-seq data, we mapped a promoter that was sufficient for reporter gene expression in the cerebral cortex throughout cortical development, and investigated the activity of two putative *Fezf2* enhancers. We demonstrate that a downstream enhancer, 434, is sufficient to drive expression in cortical progenitor cells while a putative upstream enhancer, 1316, is strictly active in deep-layer neurons within the cortex. Taken together, these results provide insight into the developmental programs that promote *Fezf2* expression during development of the cerebral cortex.

## Results

### *Fezf2* is dynamically expressed during development of the cerebral cortex

**Figure 1:**
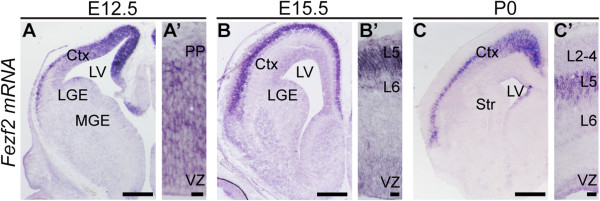
***Fezf2 *is dynamically expressed during cortical development. ***In situ* hybridization at E12.5 **(A-A’)**, E15.5 **(B-B’)**, and P0 **(C-C’)** for *Fezf2* reveals its expression pattern during development. Ctx, cortex; LGE, lateral ganglionic eminence; LV, lateral ventricle; MGE, medial ganglionic eminence; PP, preplate; Str, striatum; VZ, ventricular zone. Scale bars: **(A)** 100 μm, **(A’)** 10 μm, **(B)** 250 μm, **(B’)** 25 μm, **(C)** 500 μm, **(C’)** 50 μm.

Towards understanding how *Fezf2* transcription is regulated, we first examined its expression during cortical neurogenesis (Figure 1A-C’). Consistent with previous reports [20,21], at E12.5 *Fezf2* expression was detected in ventricular zone (VZ) progenitors and deep-layer cortical neurons (Figure 1A-A’). At E15.5 *Fezf2* expression began to decline in L6, but remained high in L5 (Figure 1B-B’). Expression at P0 was similar to E15.5 (Figure 1C-C’). Thus, at late stages of cortical development, expression of *Fezf2* in the cerebral cortex was restricted to three distinct domains: higher expression in L5 neurons, and lower expression in L6 neurons and progenitors (Figure 1C-C’). This dynamic spatial and temporal expression pattern suggests that *Fezf2* transcription is under the control of a complex regulatory program.

### Identification of putative regulatory regions around the *Fezf2* locus

**Figure 2:**
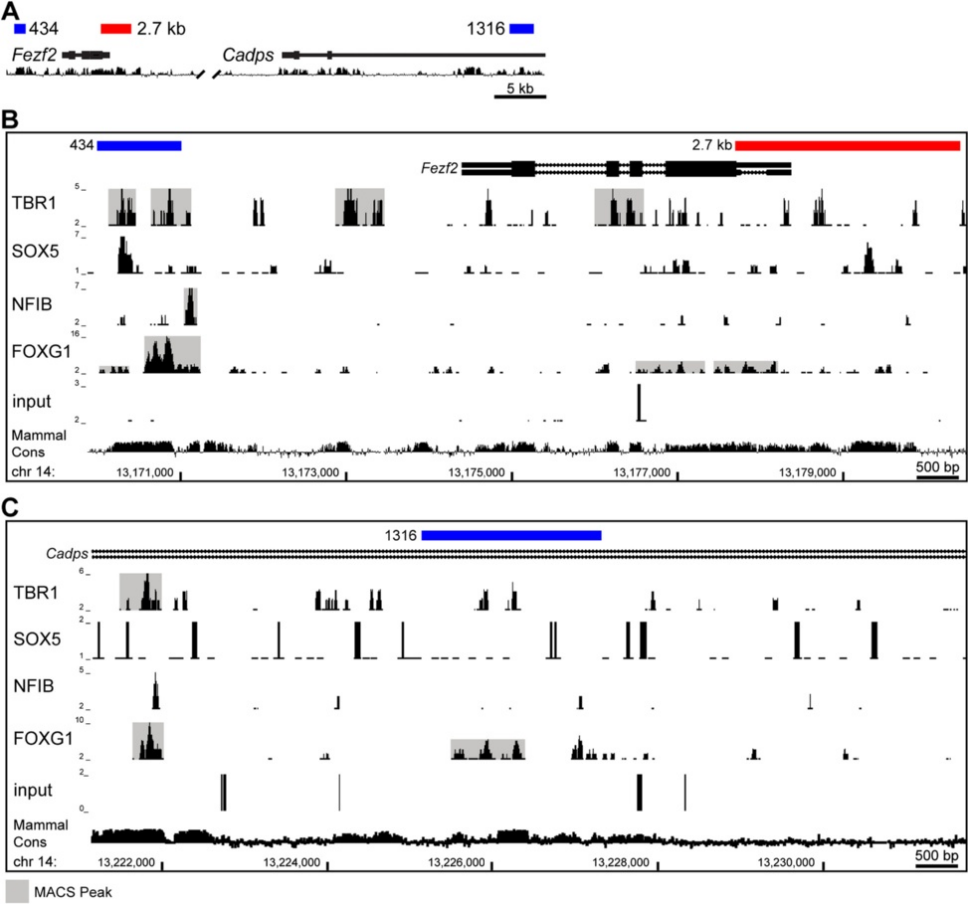
**Mapping of transcription factor binding around the *Fezf2 *locus. (A)** Diagram of the *Fezf2* locus indicating positions of the 2.7 kb promoter and the putative enhancers 434, and 1316. **(B, C)** ChIP-seq was used to map the binding of transcription factors around the *Fezf2* locus. **(B)** An overview of binding around enhancer 434 and the upstream conserved region. **(C)** An overview of factor binding around enhancer 1316. **(B, C)** Peaks called by MACS are highlighted in grey.

Recent work has identified several transcription factors that are essential for cortical neuron fate specification and differentiation. Among these, SOX5 and TBR1 appear to directly regulate *Fezf2* transcription [22-24]. Using chromatin isolated from E15.5 mouse cortices, we mapped the binding of transcription factors expressed in L5 and L6 that were previously shown to be important for cortical development. These included FOXG1, NFIB, SOX5, and TBR1 [22-33]. We found that FOXG1, SOX5, and TBR1 bound to the evolutionarily conserved region immediately upstream of *Fezf2* (Figure 2B), suggesting that this region may contain the *Fezf2* promoter. Interestingly, all of these factors bound to a previously identified enhancer downstream of *Fezf2*, enhancer 434 (also known as E4) (Figure 2B). In addition to the upstream region and enhancer 434, FOXG1 and TBR1 bound a region in the neighboring *Cadps* gene, near the 5’ end of *Fezf2* (Figure 2C). Taken together, our ChIP-seq results indicate that multiple transcription factors that are expressed in deep-layer neurons and are critical for cortical development bind at or near the *Fezf2* locus.

To determine the significance of our ChIP-seq data we searched the VISTA Enhancer Browser (http://enhancer.lbl.gov) for sequences in and near *Fezf2* that may regulate its expression [34]. This approach uncovered several enhancers around *Fezf2*. These included an enhancer at the 5’ side of *Fezf2*, enhancer 1316, and the previously studied enhancer at the 3’ side, enhancer 434 (Figure 2A). Our ChIP-seq data demonstrated binding within or near these enhancers, suggesting they are likely important for regulating *Fezf2* transcription within the cerebral cortex (Figure 2B,C).

### The 2.7 kb promoter is active across all *Fezf2* expression domains

**Figure 3:**
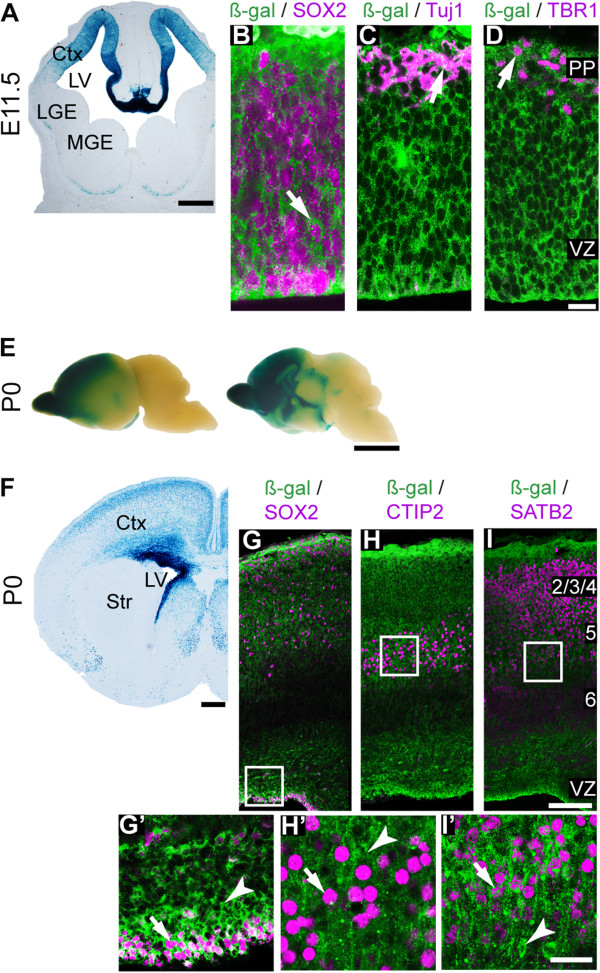
**Promoter activity of the 2.7 kb fragment during cortical development. (A)** X-Gal staining at E11.5 revealed reporter activity in the cerebral cortex, similar to endogenous *Fezf2* expression. **(B-D)** Immunohistochemistry with ß-Gal antibody combined with SOX2 **(B)**, Tuj1 **(C)**, and TBR1 **(D)** antibodies. **(E)** Whole mount X-Gal staining of P0 brains demonstrating expression in the forebrain. **(F)** X-Gal staining of P0 brain sections. **(G-I’)** Immunohistochemistry using ß-Gal antibody combined with antibodies for SOX2 **(G, G’)**, CTIP2 **(H, H’)** and SATB2 **(I, I’)**. Arrows represent co-expression of LacZ and the indicated markers. Arrowheads indicate a lack of co-expression. PP, preplate. Scale bars: **(A)** 200 μm, **(D)** 50 μm, **(E)** 250 mm, **(F)** 500 μm, **(I)** 100 μm, **(I’)** 50 μm.

Previous work identified the 2.7 kb region upstream of and including the *Fezf2* start codon as sufficient to drive forebrain expression before the onset of cortical neurogenesis [35]. This region was bound by multiple transcription factors in our ChIP-seq dataset (Figure 2B). We generated stable transgenic lines expressing the LacZ reporter under the control of this 2.7 kb sequence to study its activity throughout cortical development. When the earliest-born cortical projection neurons were being generated at E11.5, we observed LacZ activity throughout the cortex that mimicked endogenous *Fezf2* expression (compare Figure 3A with Figure 1A). We observed ß-gal expression in SOX2^+^ cortical progenitor cells and in TBR1^+^ and Tuj1^+^ postmitotic neurons (Figure 3B-D).

We next examined 2.7 kb promoter activity at P0. Whole mount X-Gal staining revealed expression throughout the cerebral cortex in a high anterior-medial to low posterior-lateral pattern (Figure 3E). However, unlike endogenous *Fezf2*, which is expressed in the VZ and in deep cortical layers (L5 and L6), the 2.7 kb-LacZ transgene was active in all cortical layers (compare Figure 3F with Figure 1C). LacZ was expressed in SOX2^+^ progenitor cells in the VZ and in the CTIP2^+^ or SATB2^+^ projection neurons (Figure 3G-I’). LacZ expression at P12 was similar to that observed at P0 (Additional file 1). Taken together, these results indicate that the 2.7 kb promoter is active throughout cortical development and is sufficient to drive transcription across all endogenous *Fezf2* expression domains (progenitor cells, L5 and L6 neurons). However, its expression in upper cortical layers indicates that additional *cis-*elements are required to properly coordinate *Fezf2* expression.

### Enhancer 434 activity is strongest in cortical progenitor cells

Previous work has shown that enhancer 434 is highly conserved across vertebrate species and is necessary for *Fezf2* expression in the cortex [36]. Deletion of this enhancer from either a bacterial artificial chromosome (BAC) containing a *Fezf2-EGFP* reporter or from its endogenous locus resulted in a loss of EGFP or *Fezf2* expression in the cerebral cortex [36]. However, it remains unknown whether enhancer 434 alone is sufficient to promote *Fezf2* expression in subcerebral projection neurons.

**Figure 4:**
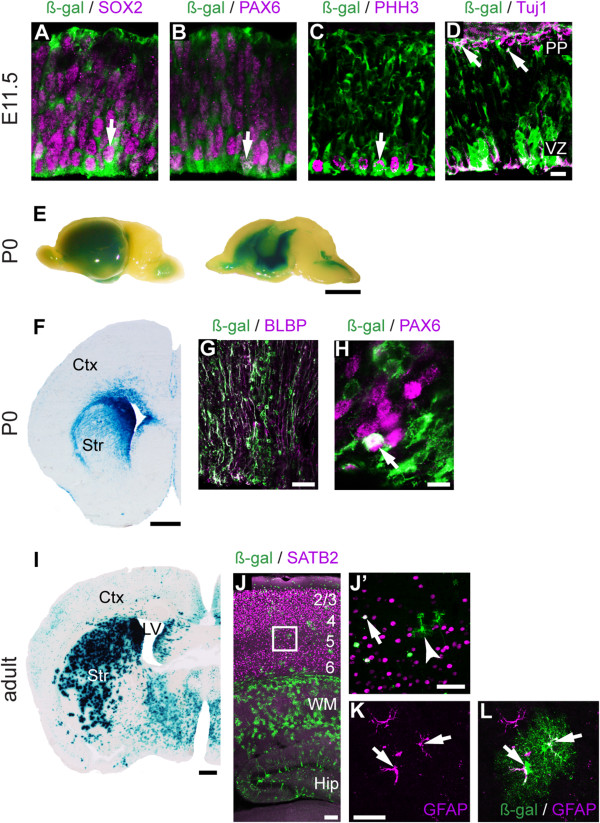
**Enhancer 434 is active in cortical progenitor cells. (A-C)** Immunohistochemistry demonstrated LacZ expression in progenitor cells at E11.5 that co-expressed SOX2 **(A)**, PAX6 **(B)** and PHH3 **(C)**. LacZ was also expressed in a few postmitotic neurons in the preplate marked by Tuj1 **(D)**. X-Gal staining of whole mount P0 brains **(E)** and sections **(F)** demonstrated reporter activity in the VZ. **(G, H)** Immunohistochemistry using ß-Gal antibody combined with antibodies for RGC markers BLBP **(G)** and PAX6 **(H)**. **(I, K, L)** In adult animals, the majority of enhancer 434-LacZ^+^ cells were in GFAP^+^ astrocytes throughout the forebrain. A subset of non-astrocyte like cells expressed SATB2 **(J, J’)**. Arrows represent co-expression of LacZ and the indicated markers. Hip, hippocampus; WM, white matter. Scale bars: **(D)** 50 μm, **(E)** 250 mm, **(F)** 500 μm, **(G)** 25 μm, **(H)** 10 μm, **(I)** 500 μm, **(J)** 100 μm, **(J’)** 50 μm, **(K)** 25 μm.

We generated stable transgenic lines expressing LacZ under the control of enhancer 434 and the hsp68 minimal promoter [37]. At E11.5, ß-gal was expressed in SOX2^+^ and PAX6^+^ cortical progenitor cells (Figure 4A-B), including the metaphase cells that were labeled by PHH3 antibody at the ventricular surface (Figure 4C). In addition, Tuj1^+^ neurons located in the preplate expressed ß-gal (Figure 4D). Whole mount staining of P0 brains revealed strong LacZ activity throughout multiple brain regions (Figure 4E). However, the majority of enhancer 434 activity was in the VZ of the cortex and basal ganglia (Figure 4F). Immunohistochemistry with antibodies for LacZ and the radial glial cell (RGC) marker BLBP demonstrated significant co-localization (Figure 4G). Additionally, some LacZ^+^ cells in the VZ expressed the RGC marker PAX6 (Figure 4H). Some cells in the cortical plate expressed LacZ, however a minority of these LacZ^+^ cells expressed the projection neuron markers SOX5 and SATB2 (Additional file 2). Thus, at P0 enhancer 434 activity is strongest in cortical progenitor cells. This strong LacZ signal observed in the VZ is consistent with the activity of enhancer 434 reported by the VISTA Enhancer Browser (http://enhancer.lbl.gov).

In adult mice, LacZ expression was observed in the cerebral cortex, white matter, hippocampus, and striatum (Figure 4I-L). Most of the LacZ^+^ cells exhibited typical astrocyte-like morphology and expressed the astrocyte marker GFAP (Figure 4K, L). A minority of LacZ^+^ cells in the cerebral cortex expressed the cortical projection neuron marker SATB2 (Figure 4J’). This shift in enhancer 434 activity suggests that it may have divergent functions during development *versus* in adult animals. Taken together, these data demonstrate that during cortical development enhancer 434 activity is strongest in progenitor cells, one of the endogenous expression domains of *Fezf2*.

### Enhancer 1316 is active in deep-layer projection neurons in the cerebral cortex

**Figure 5:**
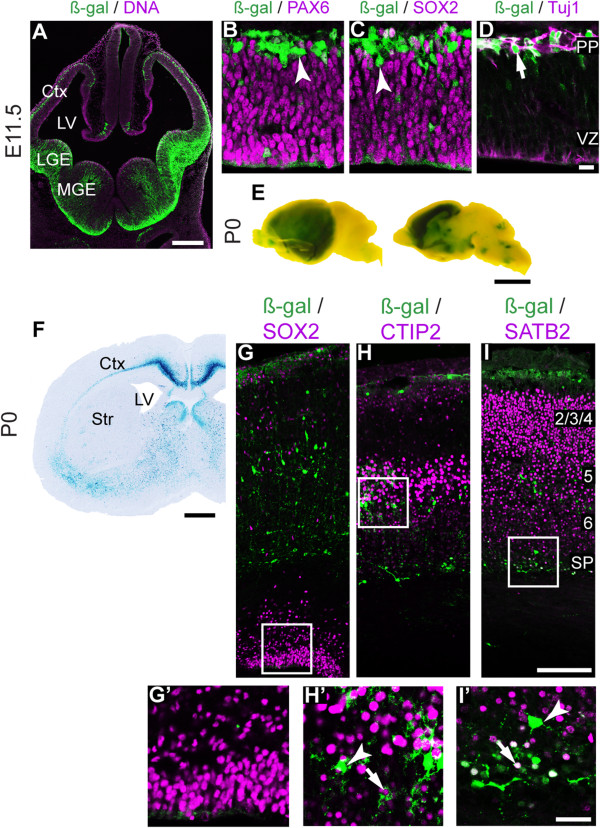
**Enhancer 1316 is active in deep-layer cortical neurons. (A-D)** Immunohistochemical analysis of E11.5 brain sections using antibodies for ß-Gal **(A)**, ß-Gal and PAX6 **(B)**, ß-Gal and SOX2 **(C)**, and ß-Gal and Tuj1 **(D)**. **(E, F)** X-Gal staining of P0 whole mount brains **(E)** and brain sections **(F)**. **(G-I) **Immunohistochemistry using ß-Gal antibody combined with antibodies for SOX2 **(G)**, CTIP2 **(H)**, and SATB2 **(I)**. Arrows represent co-expression of LacZ and the indicated markers. Arrowheads indicate a lack of co-expression. Scale bars: **(A)** 200 μm, **(D)** 50 μm, **(E)** 250 mm, **(F)** 500 μm, **(I)** 100 μm, **(I’)** 25 μm.

We next investigated the activity of enhancer 1316. MultiZ alignment of this region showed strong vertebrate conservation, especially within the middle 500 bp (Additional file 3). We generated stable transgenic lines expressing LacZ under control of enhancer 1316 and the hsp68 minimal promoter. At E11.5, ß-gal was present throughout the newly generated preplate (Figure 5A), in Tuj1^+^ neurons (Figure 5D). LacZ^+^ cells did not express the progenitor cell markers PAX6 or SOX2 (Figure 5B, C).

Whole mount staining at P0 revealed LacZ activity in deep layers of the cerebral cortex (Figure 5E). LacZ was expressed in the subplate and in some deep-layer neurons, but not in cortical progenitors or astrocytes (Figure 5F). Some LacZ^+^ cells expressed CTIP2 and SATB2 (Figure 5H-I’). 'None of the LacZ^+^ cells expressed SOX2 (Figure 5G, G’). Analysis at P12 revealed a similar LacZ expression pattern to that observed at P0 (Additional file 4). Taken together, these results indicate that within the cerebral cortex, enhancer 1316 is strictly active in deep-layer neurons.

## Discussion

**Figure 6:**
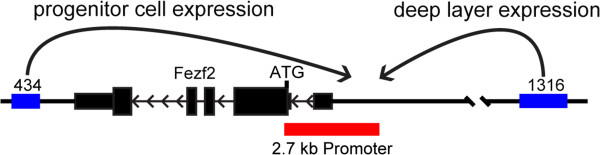
**A model for the regulation of *Fezf2 *transcription in the cortex.** The 2.7 kb promoter was sufficient to drive transcription across all endogenous *Fezf2* expression domains throughout development of the cerebral cortex. Enhancer 434 exhibited strongest activity in cortical progenitor cells and in contrast enhancer 1316 was strictly active in deep-layer postmitotic neurons.

We set out to understand how expression of *Fezf2* is spatially and temporally controlled during development of the cerebral cortex. Previous work demonstrates that *Fezf2* transcription levels are critically important for specification of cortical projection neuron fates [13-17,22-24,31]. Our ChIP-seq data indicate that multiple transcription factors that are expressed in deep-layer neurons bind to evolutionarily conserved sequences around the *Fezf2* locus. The large number of transcription factors that bound enhancer 434 underscores its developmental importance. Recent deletion analysis demonstrated that this enhancer is critical for subcerebral projection neuron identity and connectivity [36]. In the current study, the majority of enhancer 434 activity during cortical neurogenesis was in progenitor cells, suggesting that this element also functions to promote *Fezf2* expression in the VZ, one of the endogenous *Fezf2* expression domains (Figure 6).

An alternative interpretation of this result is that different experimental strategies may account for the somewhat distinct conclusions regarding the activity of enhancer 434. In the study by Shim et al. [36], enhancer 434 was deleted from either a bacterial artificial chromosome containing a *Fezf2-EGFP* reporter or from its endogenous genomic locus. In both cases, deletion of enhancer 434 resulted in a loss of GFP or *Fezf2* expression, respectively, and failed extension of subcerebral projections. In the present study, we assayed the activity of enhancer 434 in isolation and found that it drove LacZ activity strongest in progenitor cells with lower levels of reporter activity in postmitotic neurons. These data indicate that when taken out of its endogenous locus, enhancer 434 is sufficient to drive reporter expression in the progenitor cells. It is important to note however that both the Shim et al. study [36] and the data presented here indicate a critical role for enhancer 434 in the regulation of *Fezf2* transcription.

Although the present study identifies conserved regulatory elements around the *Fezf2* locus, functional enhancer-promoter interactions cannot be established based upon transgenic reporter assays alone. Specifically, whether enhancer 1316 functionally interacts with the *Fezf2* promoter to regulate transcription remains unknown. Recently published high throughput chromosome conformation capture (Hi-C) suggests an interaction between enhancer 1316 and the *Fezf2* promoter [38]. However, this enhancer does not appear to be essential for *Fezf2* expression during development. Transgenic mice containing a modified BAC (RP23-141E17) that express EGFP or CRE from the *Fezf2* open reading frame have been generated. Although enhancer 1316 is not contained within this BAC, GFP, or CRE expression in these transgenic mice recapitulates endogenous Fezf2 gene expression [36,39,40], suggesting that this enhancer is not essential for *Fezf2* expression during development. Thus, it is possible that additional enhancer(s) present on the RP23-141E17 BAC may function redundantly with enhancer 1316 to promote *Fezf2* expression in deep layer neurons.

Multiple *cis*-regulatory sequences around *Fezf2* exhibited distinct yet overlapping activities during cortical development. These results are similar to the regulation of the *eve* gene locus in *Drosophila* where multiple, partially redundant enhancers control expression in individual stripes along the anterior-posterior axis of the embryo [41,42]. Ultimately, combined activity of these enhancers is required to drive the precise spatio-temporal expression of *eve.* Similar regulatory paradigms have also been uncovered in mammals through analysis of the interferon-beta [43] or *Fgf8*[44] loci. In our study, neither of the identified enhancers nor the 2.7 kb promoter, when assayed in isolation, were sufficient to drive LacZ reporter expression in an identical pattern to that of endogenous *Fezf2* expression. This suggests the presence of an additional enhancer (enhancers) that promotes high levels of expression specifically in L5 neurons. Moreover, the strong activity of the 2.7 kb promoter in upper cortical layers indicates that repressor sequences are necessary to restrict its activity to cortical progenitor cells and deep-layer neurons. Identification of these regulatory sequences combined with an increased understanding of the chromatin environment surrounding the *Fezf2* locus should help to shed light on the mechanisms that precisely regulate its transcription.

We recently reported that in addition to deep-layer cortical neurons, *Fezf2* is expressed in progenitor cells throughout cortical neurogenesis [39]. Phenotypic analysis of *Fezf2*^*−/−*^ mice indicates that it is required for both the patterning and maintenance of forebrain progenitors [45], and the fate specification of deep-layer cortical projection neurons [13-16]. In *Fezf2*^−/−^ mutant cortices, subcerebral projection neurons switch their identity to become callosal or corticothalamic projection neurons. However, it remains unclear whether *Fezf2* functions within cortical progenitor cells, postmitotic neurons or a combination of both in order to properly specify the fate of deep-layer cortical projection neurons. Identifying distinct promoter and enhancer sequences that regulate *Fezf2* transcription may help provide answers to this question.

## Conclusions

Given recent progress identifying putative mammalian enhancers [37], it is critical that we understand the spatial and temporal activities of these sequences in the regulation of individual genes, in order to appreciate the deleterious effects of mutations in non-coding sequences identified through GWAS. Focusing on *Fezf2*, a key regulator of cortical development and deep-layer projection neuron fate, our study suggests that multiple enhancer and promoter activities can coordinate gene expression during brain development. Recent work indicates that *Fezf2* is expressed in neocortical progenitors throughout development. Although mice lacking *Fezf2* exhibit developmental defects largely within deep-layer neurons (L5 and L6), its role in cortical progenitors *versus* postmitotic neurons remains unclear. Identifying the regulatory mechanisms that control *Fezf2* transcription will enable the functional dissection of its role within these distinct cellular populations. We propose that the combined activities of the 2.7 kb promoter, enhancer 434, and possibly enhancer 1316 constitute a *cis*-regulatory module that controls *Fezf2* in distinct domains of the cerebral cortex, ultimately leading to its dynamic expression during cortical development (Figure 6).

## Methods

### Animals

All experiments in this study were carried out in accordance with protocols approved by the IACUC at the University of California, Santa Cruz and were performed in accordance with institutional and federal guidelines. The day of vaginal plug detection was designated as embryonic day 0.5 (E0.5). The day of birth was designated as postnatal day 0 (P0). Transgenic mice were generated using standard protocols [46]. To generate stable transgenic lines, founders were outcrossed to CD1 wild-type females for a minimum of three generations before analysis. Number of founder lines generated: 2.7 kb-*LacZ* (2), enhancer 434-*LacZ* (3), enhancer 1316-*LacZ* (4).

### Plasmids

Generation of the 2.7 kb-*lacZ* plasmid was previously described [35]. For enhancer 434-*lacZ*, the genomic region was obtained from mouse genomic DNA by PCR using primers: ATCGCTCGAGCAGGCTGTAGGATGGGCAGCAGGAGTTTC and ATCGAAGCTTGTAACAAGTCAGGTGAGCAGGCGGTA. The product was then cloned into the hsp68-*lacZ* expression vector using Gateway cloning according to the manufacturer’s protocol [11]. Enhancer 1316-*lacZ* was generated from human genomic DNA using primers AAACCACACAGCTGGTTTCC and TTTCCCGATAGATCGTCAGC and cloned into the hsp68-*lacZ* expression vector [37].

### Histology

Immunostaining and *in situ* hybridization were performed as previously described [47]. The *Fezf2* cRNA probe corresponds to nucleotides 644 to 1,374 of mouse *Fezf2* (GenBank: NC_000080). LacZ staining was preformed according to standard protocols. Primary antibodies used: Chicken anti β-gal (Abcam, 1:500), Rabbit anti BLBP (Millipore, 1:500), Rat anti CTIP2 (Abcam, 1:1,000), Guinea Pig anti GFAP (Advaned Immuno Chemical, 1:100), Rabbit anti PAX6 (Covance, 1:100), PHH3 (Cell Signaling, 1:100), Rabbit anti SATB2 (Abcam, 1:1,000), Goat anti SOX2 (Santa Cruz Biotech, 1:500), Rabbit anti SOX5 (Abcam, 1:500), Rabbit anti TBR1 (Abcam, 1:1,000), Mouse anti Tuj1 (Covance, 1:1,000). Primary antibodies were detected using AlexaFluor-conjugated secondary antibodies (Invitrogen, 1:1,000). DNA was visualized with DAPI (1:50,000).

### Microscopy

Bright field and epiflourescence images were captured on an Olympus BX51 microscope using a Q Imaging Retiga EXj camera or a Keyence BZ-9000 microscope. Confocal images were captured on a Leica TCS SP5 confocal microscope. Images were processed using Adobe Photoshop CS5 to adjust brightness and contrast.

### ChIP-Seq

Chromatin immunoprecipation was performed as previously described [24]. Antibodies used were Rabbit anti FOXG1 (Cell Signaling), Rabbit anti NFIB (Active Motif), Rabbit anti SOX5 (Abcam), and Rabbit anti TBR1 (Abcam). Sequencing libraries were generated using the Illumina TruSeq kit according to the manufacturer’s protocol. Sequencing was performed on an Illumina Hiseq 2000 at the UCSC Genome Technology Center. Input DNA was sequenced as control. Sequencing reads were mapped to the mouse genome (mm9) using the Bowtie mapping algorithm. Non-overlapping reads and PCR duplicates were removed. Peaks were called using the MACS algorithm [48].

## Abbreviations

BAC: Bacterial artificial chromosome; ß-gal: ß-galactosidase; ChIP-seq: Chromatin co-immunoprecipitation and high throughput DNA sequencing; E: Embryonic day; GWAS: Genome-wide association study; L5: Layer 5; L6: Layer 6; P: Postnatal day; PCR: Polymerase chain reaction; RGC: Radial glial cell; SCPN: Subcerebral projection neurons; VZ: Ventricular zone.

## Competing interests

The authors declare that they have no competing interests.

## Authors’ contributions

MJE: conception and design, data collection and analysis, manuscript writing, and final approval of the manuscript. KAL: data collection and analysis, final approval of the manuscript. WLM: data collection and analysis, manuscript writing, and final approval of the manuscript. CG: conception and design, data collection, manuscript writing, and final approval of the manuscript. RR: data collection and final approval of the manuscript. SK: analyzing sequencing data and final approval of the manuscript. AV: generating the enhancer 1316-LacZ reporter construct, manuscript editing, and final approval of the manuscript. JLRR: generating the enhancer 1316-LacZ reporter construct, manuscript editing, and final approval of the manuscript. BC: conception and design, data analysis, manuscript writing, and final approval of the manuscript. All authors read and approved the final manuscript.

## Supplementary Material

Additional file 1 **2.7 kb promoter activity at P12. (A)** The activity of the 2.7 kb promoter at P12 was similar to that observed at P0, with expression throughout the cortex and the strongest activity in upper layers. **(B-C’)** LacZ was expressed in some CTIP2^+^ and TBR1^+^ cells in deep layers. **(D, D’)** However, the highest density of LacZ^+^ cells was in upper layers cells that expressed SATB2. Panels B’-D’ show amplified areas boxed in panels B-D, respectively. Arrows represent co-expression of LacZ and the indicated markers. Arrowheads indicate a lack of co-expression. Ctx, cortex; LV, lateral ventricle; Str, striatum. Scale bars: (a) 500 μm, (d) 100 μm, (d’) 50 μm. Click here for file

Additional file 2 **Enhancer 434 activity at P0. (A-B’)** LacZ expression was observed in a few post-mitotic neurons expressing SOX5 **(A, A’)** or SATB2 **(B, B’)**. A’ and B’ show amplified areas boxed in panels A and B. Arrows represent co-expression of LacZ and the indicated markers. Arrowheads indicate a lack of co-expression. Scale bars: (B) 100 μm, (B’) 50 μm. Click here for file

Additional file 3 **Vertebrate conservation of enhancer 1316.** The UCSC genome browser was used to perform Multiz alignment of enhancer 1316 from 30 vertebrates. Strong conservation was most evident within the middle 500 bp (approximately) of this region. Basewise conservation is represented as greyscale darkness with higher conservation corresponding to darker values. Gap Annotation: single line, no bases in the aligned species; double line, aligning species has one or more un-alignable bases in the gap region; red coloring, aligning species has Ns in the gap region. Genomic Breaks: green square brackets, enclose shorter alignments consisting of DNA from one genomic context in the aligned species nested inside a larger chain of alignments from a different genomic context. Click here for file

Additional file 4 **Activity of enhancer 1316 at P12. (A-D’)** Expression at P12 mirrored P0. (A) X-Gal staining. **(B-D’)** Immunohistochemistry analysis of the brain sections. Some LacZ positive cells in deep layers expressed CTIP2 (B-B’), SATB2 (C-C’), and TBR1 (D-D’). B’-D’ show enlargement of areas boxed in panels B-D. Ctx, cortex; LV, lateral ventricle; Str, striatum. Scale bars: (A) 500 μm, (D) 100 μm, (D’) 20 μm. Click here for file
