# Supplementary figures and images for: Multiple conserved regulatory domains promote Fezf2 expression in the developing cerebral cortex
Source: Neural Dev. 2014 Mar 12;9:6. doi: 10.1186/1749-8104-9-6 (PMC4008173; doi:10.1186/1749-8104-9-6)

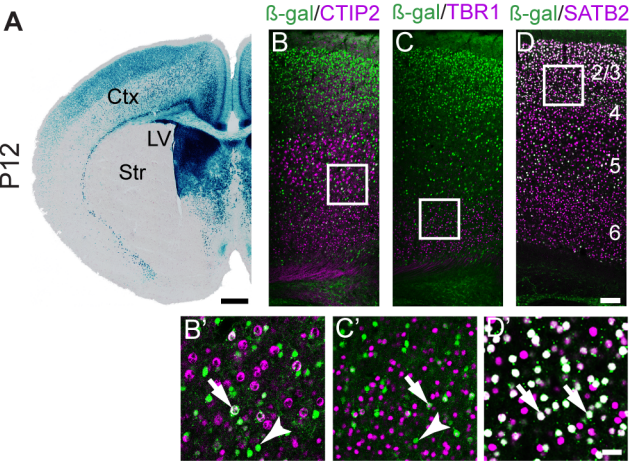

Supplement: Additional file 1 — 2.7 kb promoter activity at P12. (A) The activity of the 2.7 kb promoter at P12 was similar to that observed at P0, with expression throughout the cortex and the strongest activity in upper layers. (B-C’) LacZ was expressed in some CTIP2+ and TBR1+ cells in deep layers. (D, D’) However, the highest density of LacZ+ cells was in upper layers cells that expressed SATB2. Panels B’-D’ show amplified areas boxed in panels B-D, respectively. Arrows represent co-expression of LacZ and the indicated markers. Arrowheads indicate a lack of co-expression. Ctx, cortex; LV, lateral ventricle; Str, striatum. Scale bars: (a) 500 μm, (d) 100 μm, (d’) 50 μm. [file 1749-8104-9-6-S1.pdf]

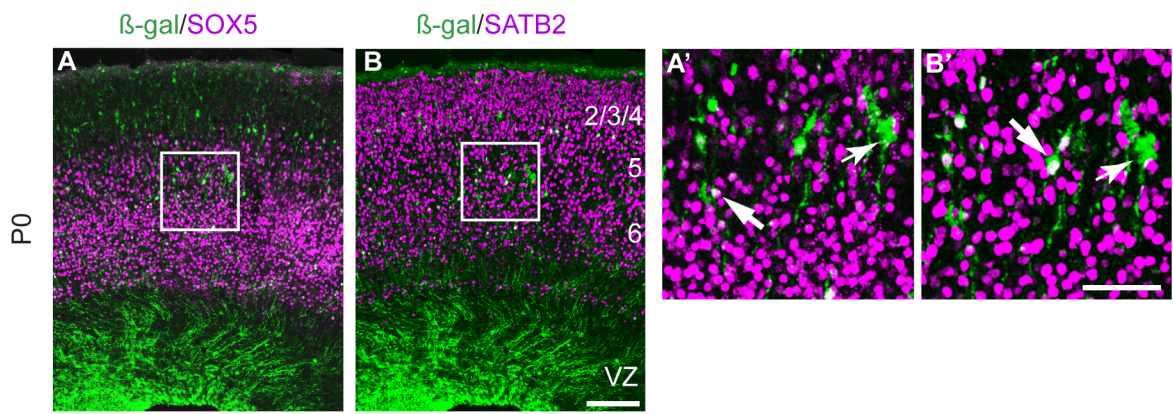

Supplement: Additional file 2 — Enhancer 434 activity at P0. (A-B’) LacZ expression was observed in a few post-mitotic neurons expressing SOX5 (A, A’) or SATB2 (B, B’). A’ and B’ show amplified areas boxed in panels A and B. Arrows represent co-expression of LacZ and the indicated markers. Arrowheads indicate a lack of co-expression. Scale bars: (B) 100 μm, (B’) 50 μm. [file 1749-8104-9-6-S2.pdf]

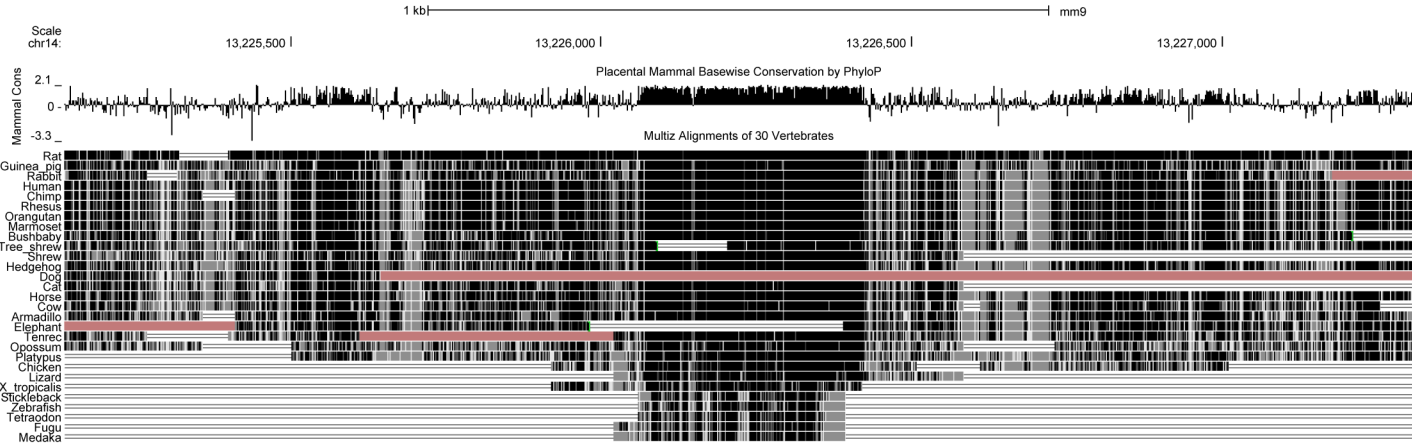

Supplement: Additional file 3 — Vertebrate conservation of enhancer 1316. The UCSC genome browser was used to perform Multiz alignment of enhancer 1316 from 30 vertebrates. Strong conservation was most evident within the middle 500 bp (approximately) of this region. Basewise conservation is represented as greyscale darkness with higher conservation corresponding to darker values. Gap Annotation: single line, no bases in the aligned species; double line, aligning species has one or more un-alignable bases in the gap region; red coloring, aligning species has Ns in the gap region. Genomic Breaks: green square brackets, enclose shorter alignments consisting of DNA from one genomic context in the aligned species nested inside a larger chain of alignments from a different genomic context. [file 1749-8104-9-6-S3.pdf]

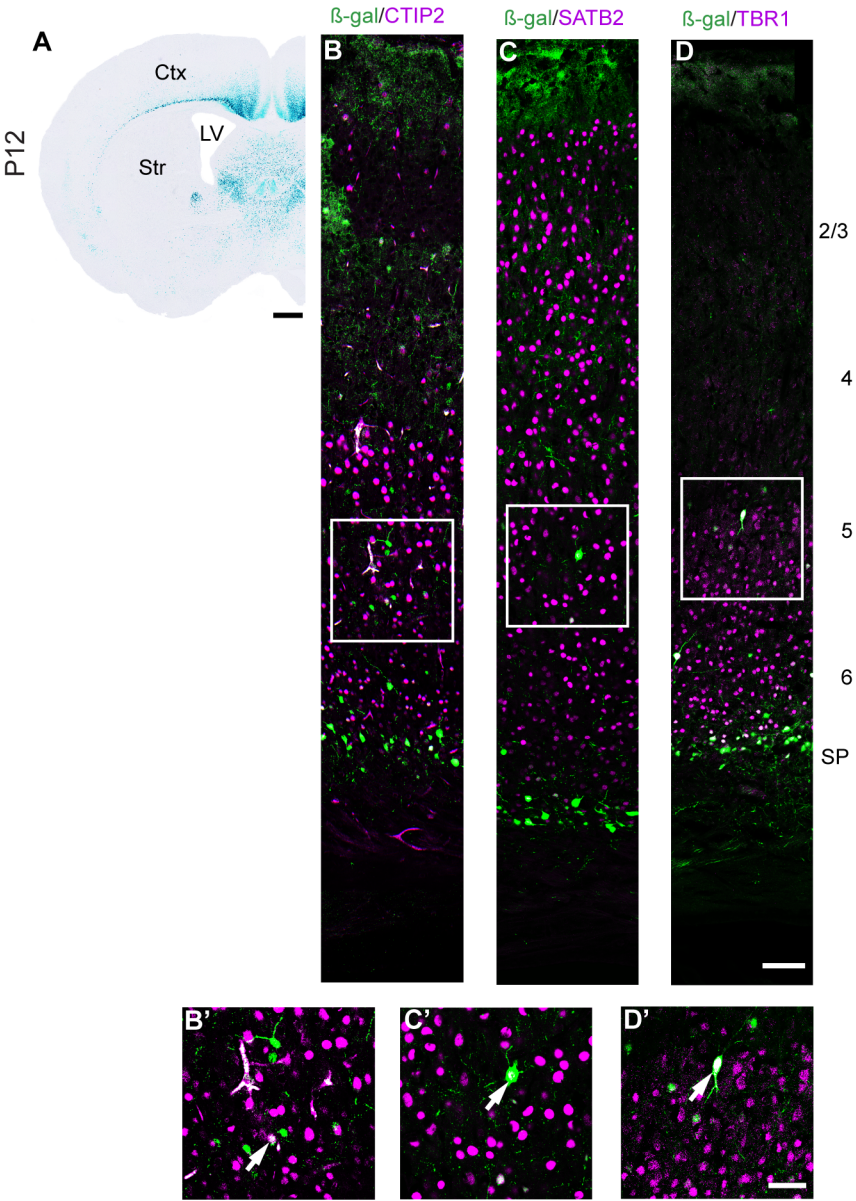

Supplement: Additional file 4 — Activity of enhancer 1316 at P12. (A-D’) Expression at P12 mirrored P0. (A) X-Gal staining. (B-D’) Immunohistochemistry analysis of the brain sections. Some LacZ positive cells in deep layers expressed CTIP2 (B-B’), SATB2 (C-C’), and TBR1 (D-D’). B’-D’ show enlargement of areas boxed in panels B-D. Ctx, cortex; LV, lateral ventricle; Str, striatum. Scale bars: (A) 500 μm, (D) 100 μm, (D’) 20 μm. [file 1749-8104-9-6-S4.pdf]
